# Co-Mutation of FAT3 and LRP1B in Lung Adenocarcinoma Defines a Unique Subset Correlated With the Efficacy of Immunotherapy

**DOI:** 10.3389/fimmu.2021.800951

**Published:** 2022-01-06

**Authors:** Mingyu Zhu, Lu Zhang, Haiyan Cui, Qiang Zhao, Hao Wang, Baochao Zhai, Richeng Jiang, Zhansheng Jiang

**Affiliations:** ^1^ Tianjin Medical University Cancer Institute and Hospital, National Clinical Research Center for Cancer, Tianjin, China; ^2^ Key Laboratory of Cancer Prevention and Therapy, Tianjin, China; ^3^ Tianjin’s Clinical Research Center for Cancer, Tianjin, China; ^4^ Cancer Precise Diagnosis Center, Tianjin Cancer Hospital Airport Hospital, Tianjin, China; ^5^ Center for Precision Cancer Medicine & Translational Research, Tianjin Medical University Cancer Institute and Hospital, Tianjin, China; ^6^ Department of Breast Oncology, Tianjin Cancer Hospital Airport Hospital, Tianjin, China; ^7^ Medical Affairs Office, Tianjin Cancer Hospital Airport Hospital, Tianjin, China; ^8^ Department of Integrative Oncology, Tianjin Medical University Cancer Institute and Hospital, Tianjin, China

**Keywords:** FAT3, LRP1B, co-mutation, lung adenocarcinoma, immunotherapy

## Abstract

Immunotherapy based on immune checkpoint inhibitors (ICIs) have demonstrated remarkable survival benefits and gained regulatory approval in non-small cell lung cancer (NSCLC) patients without an actionable driver mutation, but currently there is no well-established standard for how to screen the most suitable population for ICIs treatment. Here, we conducted a comprehensive analysis of the somatic mutation landscape of lung adenocarcinoma (LUAD) samples. After the stepwise screening of high-frequency mutated genes, two genes with prominent significance, FAT3 and LRP1B, were finally screened out. Through further analysis, we discovered that the co-mutation of FAT3 and LRP1B was associated with an earlier age of onset and occurred more frequently in Black/African American. Furthermore, co-mutation defines a unique subgroup of lung adenocarcinoma that can increase tumor mutational burden (TMB), boost cytotoxicity and tumor immunogenicity, and facilitate lymphocyte infiltration. The results of gene set enrichment analysis (GSEA) indicated that co-mutation can influence tumorigenesis through a variety of mechanisms. More strikingly, the subset of LUAD with co-mutation of FAT3 and LRP1B exhibited significantly prolonged immunotherapy progression free survival (PFS). In summary, co-mutation of FAT3 and LRP1B is a promising useful biomarker for predicting the efficacy of immunotherapy, which can improve the clinical efficiency of practicing precision medicine in lung adenocarcinoma patients.

## Introduction

Lung cancer is currently the leading cause of cancer death and second most diagnosed cancer worldwide, with an incidence of 11.4% and a mortality far higher than any other cancer types (18.0%) (1). Approximately 1.8 million patients died of lung cancer and 2.2 million new lung cancer cases globally in 2020 (1). Despite great progress have been made in the treatment of lung cancer, the five-year survival rate for patients diagnosed between 2010 and 2014 was only 10% to 20% in most countries (2). The prevalence of lung cancer in China has been among the top for many years. In 2015, lung cancer was the most common malignancy and caused the most cancer-related deaths in China (3). Adenocarcinoma is the most common histologic subtype of lung cancer, belonging to NSCLC (4). In recent years, immunotherapy based on monoclonal antibodies targeting immune checkpoint programmed cell death-1 (PD-1) and programmed cell death-1 ligand (PD-L1) has achieved remarkable clinical success and shown unprecedented durable responses for NSCLC patients without an actionable driver mutation (5–8). The emergence of ICIs has become an exciting treatment option for these patients, and has dramatically changed the way they are treated (9). Unfortunately, only a minority of NSCLC patients can really benefit from ICIs treatment (10).

PD-L1 expression, microsatellite instability high (MSI-H)/mismatch repair deficiency (dMMR) and TMB have gained regulatory approval as predictive biomarkers for ICIs in the treatment of NSCLC (5, 8, 11–13). PD-L1 expression is the first biomarker developed to enrich the population who are sensitive to PD-1/PD-L1 targeted immunotherapy. The interpretation of PD-L1 immunohistochemistry (IHC) requires professional pathologists to undergo special training to ensure the accuracy of the results. Different ICIs need to be detected by diverse antibodies, and different antibodies require distinct IHC platforms and have various positivity interpretation standards (14). MSI-H occurs when MMR proteins are dysfunctional and unable to repair errors caused by DNA replication in the microsatellite (15). MSI-H/dMMR mainly exists in colorectal cancer, endometrial cancer and gastric cancer, and its incidence in LUAD is very low (16). TMB is generally determined by whole-exome sequencing or targeted gene panel sequencing with a coding sequence (CDS) greater than or equal to 1.0Mb, and has been used as an effective indicator of response in immunotherapy for many cancer types (17). In most cancers, PD-L1 expression and TMB are two independent biomarkers, and the level of PD-L1 expression has no connection with the level of TMB (18, 19). MSI-H can be understood as a subset of TMB-H. Patients with TMB-H are not necessarily MSI-H, but most patients with MSI-H are also TMB-H (20, 21), because in addition to errors in mismatch repair, defects in other DNA damage repair pathways can also lead to increased mutation rates (22). Tumor somatic mutations can generate non-self neoantigens that confer immunogenicity and induce anti-tumor immune response (23). Therefore, mutations not only contribute to tumorigenesis and progression, but also increase the chance of tumors being recognized by the host immune system and lead to tumor elimination in the meanwhile.

It is well established that simpler methods, such as single-gene or multi-gene co-mutation detection can be used as an alternative to predict TMB (22, 24, 25). LRP1B is a tumor suppressor gene, encoding low-density lipoprotein (LDL) family receptor (26), and its mutation frequency is among the top ten in LUAD (27). The correlation of LRP1B with TMB and immunotherapy efficacy has been confirmed in multiple cancers, including lung cancer, melanoma and other solid tumors (28, 29). FAT3 belongs to the FAT family genes encoding large proteins with extracellular Cadherin repeats, EGF-like domains, and Laminin G-like domains, and is involved in tumor suppression and planar cell polarity (PCP) (30). Similarly, FAT3 mutations have been linked to prognosis and elevated TMB level in NSCLC (31), esophageal cancer (ESCA) (32) and triple-negative breast cancer (TNBC) (33). Although FAT3 gene mutations are common in many cancers, including lung adenocarcinoma, there are few studies on the association between FAT3 gene and immunotherapy.

In this study, we comprehensively analyzed the gene sequencing data of lung adenocarcinoma samples, and screened out 18 genes with non-synonymous mutations frequencies greater than 20% in the coding region. Next, we further explored the correlation between these high-frequency mutated genes and TMB, mRNA expression levels of recombinant cluster of differentiation 8A (CD8A) and interferon gamma (IFNG), neoantigen and immunotherapy benefit, and finally acquired two significantly related genes, FAT3 and LRP1B. Considering the co-occurrence relationship between these two key genes, we conducted an in-depth analysis of LUAD samples with co-mutation of FAT3 and LRP1B, performed GSEA analysis and investigated the impact of co-mutation on immune infiltration. Overall, our results demonstrate that compared with FAT3 or LRP1B single-mutation samples, the LUAD subset with co-mutation of FAT3 and LRP1B exhibit more unique clinical profiles and is more closely associated with the efficacy of immunotherapy.

## Materials and Methods

### Patient Selection

The study cohort consisted of 506 LUAD patients selected and retrieved from The Cancer Genome Atlas (TCGA, http://cancergenome.nih.gov), and the mutation and expression data from these populations were used for comprehensive integrated analysis. The cohort was obtained by screening samples with both somatic mutation and mRNA expression profiling. A total of 90 LUAD samples from Rizvi et al. (15 cases) (34) and Hellmann et al. (75 cases) (18) were used to explore the association of gene mutations with neoantigen and the efficacy of immunotherapy. In addition, 485 LUAD samples from Imielinksi et al. (183 cases) (35) and Chen et al. (302 cases) (36) were used to verify the relationship between mutation status and TMB level. Gene mutations were defined as all mutations in CDS region except synonymous and intron mutations, including indels, missense mutations, nonsense mutations and splice mutations. Given the absence of patient identification information and the retrospective nature of the study, ethical approval and informed consent was waived.

### Identification of Frequently Mutated Genes

Based on the original gene mutation results of 506 LUAD samples from TCGA, a TXT file annotated by hg19 reference genome was generated, including only three columns of sample ID, mutant gene and variant class. Among them, the variant class was composed of all mutations in CDS region except synonymous and intron mutations. The TXT file was visualized through the GenVisR package for somatic mutations, ranking mutated genes in the order of mutation frequency from high to low to obtain 18 genes with mutation frequency greater than 20% in the cohort.

### Molecular Characteristics Analysis

The total number of non-synonymous somatic variants obtained from whole exome sequencing (WES) data, divided by the size of the exome, was used as the formula to calculate the TMB of LUAD samples. All single nucleotide variants (SNVs) and indels were included. The expression of CD8A and IFNG were evaluated through transcriptome data in RNASeqV2 RSEM format, log_2_(RSEM+1) transformed. The level of neoantigen had previously been measured in the immunotherapy cohort by published methods (18, 34). By assessing the correlation of these 18 genes with TMB, the expression levels of CD8A and IFNG, and neoantigen for stepwise screening, the two key genes, FAT3 and LRP1B, which had important implications in LUAD, were finally obtained.

### Gene Set Enrichment Analysis

In order to explore the mechanism of the mutation status of target genes in the occurrence and development of LUAD tumors, we divided the TCGA cohort into two groups, FAT3 and LRP1B co-mutation and wild-type, and performed GSEA by RNA-seq data. Using GSEA software (version 4.1.0) (37), we analyzed which classic signaling pathways are affected by co-mutation of FAT3 and LRP1B. Enrichment score (ES) reflected the degree to which a gene set was overrepresented at top or bottom of the entire ranked list, and ES was normalized according to the size of gene set to yield normalized enrichment score (NES). Permutations for each analysis were set as 1000 times. Pathways with a normal p-value less than 0.05 were considered to be significantly enriched.

### Co-Mutation and Tumor-Infiltrating Lymphocytes

Based on the deconvolution algorithm CIBERSORT (38), we used RNA-seq data to evaluate the relative abundance of 22 tumor-infiltrating immune cells in the TCGA LUAD dataset. We then divided the cohort into FAT3 and LRP1B co-mutation and wild-type groups according to the mutation status of target genes to discuss the effect of co-mutation on the degree of lymphocyte infiltration. The correlation between immune cells was visualized using corrplot package and Pearson correlation coefficient was calculated.

### Statistical Analysis

Demographic, clinicopathological, and molecular characteristics were treated as either continuous (e.g., age, TMB, mRNA expression) or categorical (e.g., sex, stage, race) variables as appropriate. We used the nonparametric Mann-Whitney for all comparisons of continuous data, and categorical variables were compared using the Fisher exact test. For the analysis of the association of target gene mutations with the efficacy of ICIs treatment, survival curves were plotted using the Kaplan-Meier method and compared by the log-rank test. The PFS data were evaluated by Response Evaluation Criteria in Solid Tumors, version 1.1 (RECISTv1.1). PFS was defined as the time from the initiation of therapy to the date of disease progression or death from any cause. Statistical analyses were conducted using R (version 3.6.1), SPSS (version 25.0) and GraphPad Prism (version 8.0). All statistical tests were two-tailed, and p value under 0.05 was deemed significant.

## Results

### Somatic Mutation Landscape in Lung Adenocarcinoma

**Figure 1:**
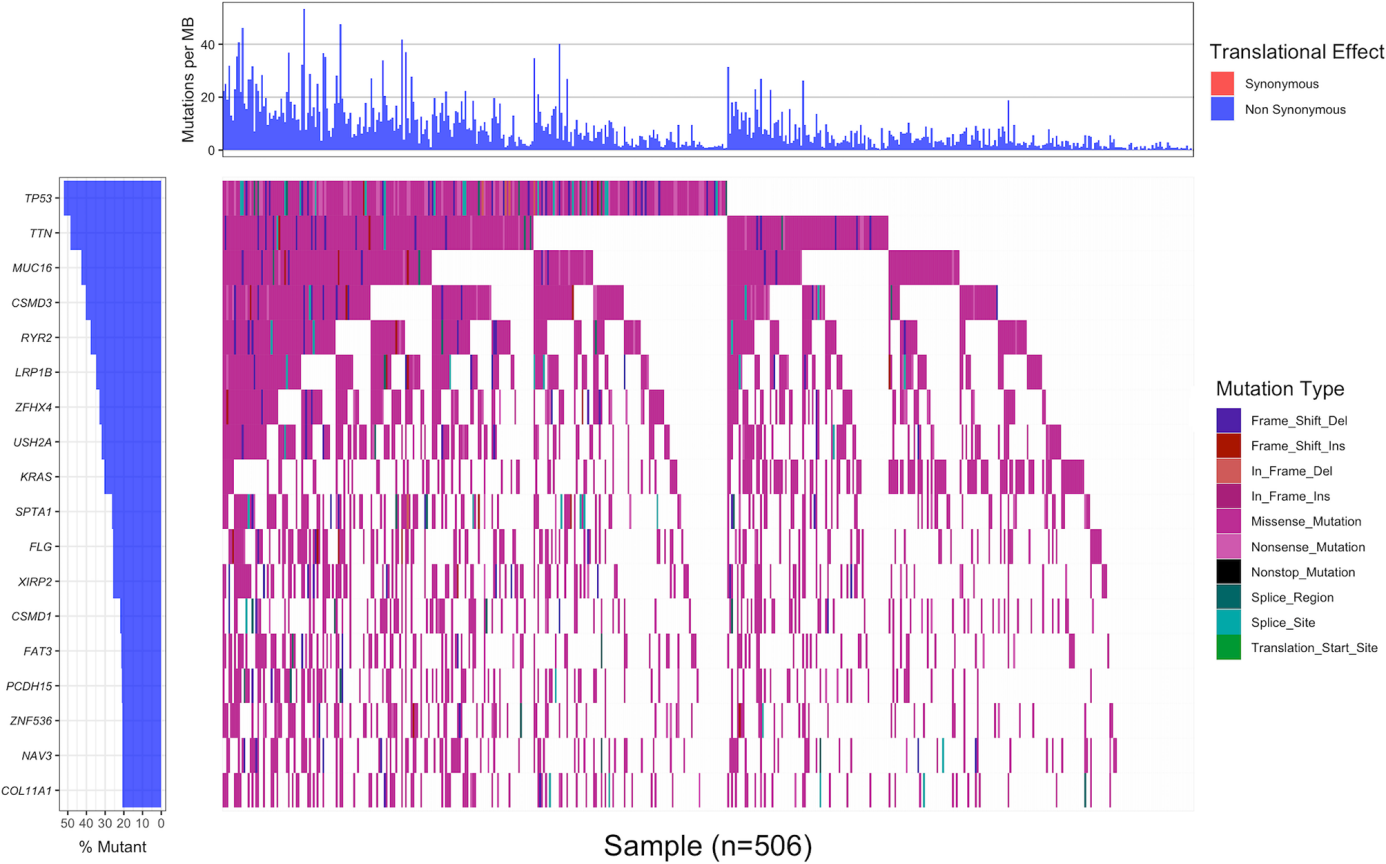
The waterfall plot displaying the landscape of frequently mutated genes in 506 LUAD samples with non-synonymous mutation frequency in the coding region greater than 20%. Genes were arranged in descending order of mutation frequency (left panel), and different colors represented different mutation types (right panel). The upper panel indicated the number of non-synonymous mutations in the coding region per Mb of each sample.

Using 20% as the cut-off value of gene mutation frequency, we screened out 18 genes (TP53, TTN, MUC16, CSMD3, RYR2, LRP1B, ZFHX4, USH2A, KRAS, SPTA1, FLG, XIRP2, CSMD1, FAT3, PCDH15, ZNF536, NAV3 and COL11A1) based on the waterfall plot of 506 LUAD samples, of which TP53 had the highest mutation frequency (52.0%, 263 of 506) (**Figure 1**). All mutations in the CDS region that might alter the amino acid sequence were included, such as indels, missense mutations, nonsense mutations and splice mutations. Missense mutation was the most common type of variation.

### The Association of Target Gene Mutations With TMB and the Expression Levels of CD8A and IFNG

**Figure 2:**
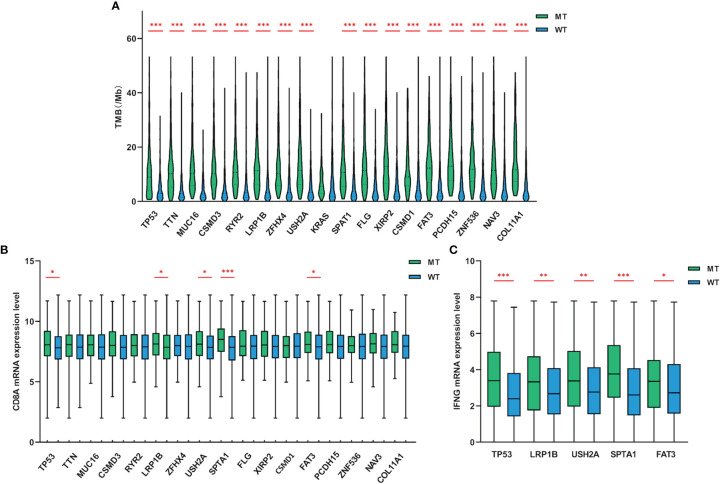
Gene mutations were related to TMB and the expression levels of CD8A and IFNG. **(A)** Compared with wild-type groups, LUAD samples with TP53, TTN, MUC16, CSMD3, RYR2, LRP1B, ZFHX4, USH2A, SPTA1, FLG, XIRP2, CSMD1, FAT3, PCDH15, ZNF536, NAV3 and COL11A1 mutations had significantly higher TMB. **(B, C)** LUAD samples with TP53, LRP1B, USH2A, SPTA1 and FAT3 mutations had higher CD8A and IFNG mRNA expression levels (adding one pseudo-count and log_2_ transformation) than wild-type. WT=wild-type. p values indicate comparisons between mutant and wild-type LUADs by Mann-Whitney test. *p < 0.05, **p < 0.01, ***p < 0.001.

TMB reflects the number of mutations contained in tumor cells, usually measured by the number of mutations per Mb in the CDS region of the genome, and has been used as an effective predictor of immunotherapy response in lung cancer (5, 8). LUAD patients with TP53, TTN, MUC16, CSMD3, RYR2, LRP1B, ZFHX4, USH2A, SPTA1, FLG, XIRP2, CSMD1, FAT3, PCDH15, ZNF536, NAV3 and COL11A1 mutations in the cohort exhibited a much higher TMB than wild-type patients (**Figure 2A**). The TMB of the dataset ranged from 0.00 to 53.29 mutations/Mb, with a median of 5.42 mutations/Mb. Further analysis indicated that regardless of the median TMB or 10 mutations/Mb as the threshold, the level of TMB had no significant correlation with the prognosis of LUAD (**Supplementary Figure 1**).

Tumors possessing higher level of TMB may stimulate the immune system to produce more potent cytotoxicity. To discover whether these target genes that were significantly related to TMB had similar impact on the tumor microenvironment (TME), we further examined the cytotoxic T-cell markers of mutant LUADs. Compared with wild-type group, the expression of CD8A (**Figure 2B**) and IFNG (**Figure 2C**) were significantly upregulated in LUAD patients with TP53, LRP1B, USH2A, SPTA1 and FAT3 mutations. Collectively, LUAD subgroups with TP53, LRP1B, USH2A, SPTA1 and FAT3 mutations harbored higher TMB level and exhibited enhanced cytotoxicity.

### Gene Mutations Are Relevant With Neoantigen Level and Immunotherapy Outcome

**Figure 3:**
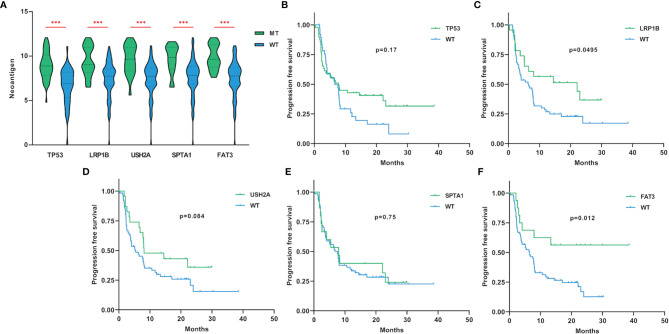
The associations of gene mutations with neoantigen and immunotherapy PFS. **(A)** LUAD samples with TP53, LRP1B, USH2A, SPTA1 and FAT3 mutations had higher levels of neoantigen (adding one pseudo-count and log_2_ transformation). **(B–F)** Compared with non-mutated LUAD samples, LUADs with LRP1B and FAT3 mutations had significantly longer PFS for immunotherapy. WT=wild-type. Statistical comparisons between different groups were made by Mann-Whitney test **(A)**, and survival curves were plotted using the Kaplan-Meier method and compared by the log-rank test **(B–F)**. ***p < 0.001.

A large number of non-synonymous mutations are thought to cause the emergence of more neoantigens, which further enhances immunogenicity and makes tumors more sensitive to ICIs treatment (13). To investigate whether higher TMB can translate into favorable immunotherapy response, we conducted a more in-depth examination of subgroups with target gene mutations. Through a dataset containing 90 LUAD samples treated with ICIs, we found that TP53, LRP1B, USH2A, SPTA1 and FAT3-mutant LUADs all had higher neoantigen loads than wild-type (**Figure 3A**). More importantly, LUADs with LRP1B (22.1 months vs 6.5 months, HR=0.55, 95% CI=0.32-0.94, p=0.0495) and FAT3 (not reached vs 6.5 months, HR=0.38, 95% CI=0.22-0.68, p=0.012) mutations also had significantly prolonged immunotherapy PFS (**Figures 3B–F**). In general, these results suggested that LUAD samples with LRP1B and FAT3 mutations possessed increased immunogenicity and better immunotherapy outcomes.

### Characteristics of Lung Adenocarcinoma Patients With FAT3 and LRP1B Mutations

Across 506 LUAD patients, the mutation frequencies of LRP1B and FAT3 genes were 21.34% (108 of 506) and 34.78% (176 of 506), respectively, and the frequency of co-mutation of FAT3 and LRP1B genes was 10.87% (55 of 506). Furthermore, co-occurrence and mutual exclusivity analysis of mutations confirmed that FAT3 and LRP1B mutations tended to occur simultaneously (Fisher’s exact test, p<0.001). Consequently, we sought to divide the LUAD cohort into four groups: FAT3 and LRP1B co-mutation group (FAT3^+^/LRP1B^+^), FAT3 mutation only group (FAT3^+^), LRP1B mutation only group (LRP1B^+^), and FAT3 and LRP1B double wild-type group (WT), to analyze the relationship between mutation status and demographic and clinicopathological characteristics of lung adenocarcinoma patients (**Table 1**). Compared with wild-type LUADs, patients in the FAT3^+^/LRP1B^+^ group were correlated with earlier cancer onset (median age 63.5 vs 67 years old, p=0.012), and the tumors were more commonly occurred in Black/African American (18.18% vs 8.30%, p=0.037). There were no significant differences between the FAT3^+^/LRP1B^+^ and WT groups in terms of sex, stage, grade and tumor status. Additionally, compared with the WT group, the FAT3^+^ and LRP1B^+^ groups had no striking differences in these characteristics.

**Table 1:** Demographic and clinicopathological characteristics in the LUAD cohort according to FAT3 and LRP1B mutation status.

	FAT3^+^/LRP1B^+^	FAT3^+^	LRP1B^+^	WT	p value	p value	p value
					(FAT3^+^/LRP1B^+^ *vs* WT)	(FAT3^+^ *vs* WT)	(LRP1B^+^ *vs* WT)
**Number**	55 (10.87%)	53 (10.47%)	121 (23.91%)	277 (54.74%)			
**Age**	63.5 (41-87)	63 (39-81)	66 (38-87)	67 (40-88)	**0.012^†^**	0.065^†^	0.181^†^
**Sex**					0.239	0.368	0.51
Female	25 (45.45%)	25 (47.17%)	71 (58.68%)	151 (54.51%)			
Male	30 (54.55%)	28 (52.83%)	50 (41.32%)	126 (45.49%)			
**Race**					**0.037**	0.178	0.564
Asian	1 (1.82%)	0	2 (1.65%)	5 (1.81%)			
White	34 (61.82%)	36 (67.92%)	93 (76.86%)	217 (78.34%)			
Black/African American	10 (18.18%)	8 (15.09%)	11 (9.09%)	23 (8.30%)			
Other	0	0	1 (0.83%)	0			
Unknown	10 (18.18%)	9 (16.98%)	14 (11.57%)	32 (11.55%)			
**Stage**					0.54	0.7	0.221
I	27 (49.09%)	26 (49.06%)	69 (57.02%)	152 (54.87%)			
II	18 (32.73%)	15 (28.30%)	21 (17.36%)	66 (23.83%)			
III	6 (10.91%)	10 (18.87%)	26 (21.49%)	41 (14.80%)			
IV	3 (5.45%)	2 (3.77%)	5 (4.13%)	17 (6.14%)			
Unknown	1 (1.82%)	0	0	1 (0.36%)			
**pT stage**					0.595	0.942	0.671
T1	17 (30.91%)	18 (33.96%)	44 (36.36%)	88 (31.77%)			
T2	29 (52.73%)	29 (54.72%)	65 (53.72%)	149 (53.79%)			
T3	8 (14.55%)	5 (9.43%)	7 (5.79%)	25 (9.03%)			
T4	1 (1.82%)	1 (1.89%)	5 (4.13%)	12 (4.33%)			
Unknown	0	0	0	3 (1.08%)			
**pN stage**					0.958	0.58	0.165
N0	37 (67.27%)	31 (58.49%)	77 (63.64%)	179 (64.62%)			
N1	12 (21.82%)	12 (22.64%)	18 (14.88%)	54 (19.49%)			
N2	6 (10.91%)	9 (16.98%)	24 (19.83%)	34 (12.27%)			
N3	0	0	1 (0.83%)	1 (0.36%)			
Unknown	0	1 (1.89%)	1 (0.83%)	9 (3.25%)			
**pM stage**					1	0.746	0.802
M0	36 (65.45%)	37 (69.81%)	82 (67.77%)	183 (66.06%)			
M1	3 (5.45%)	2 (3.77%)	5 (4.13%)	15 (5.42%)			
Unknown	16 (29.09%)	14 (26.42%)	34 (28.10%)	79 (28.52%)			
**Neoplasm Cancer Status**					0.717	0.718	1
Tumor Free	36 (65.45%)	35 (66.04%)	69 (57.02%)	164 (59.21%)			
With Tumor	11 (20.00%)	11 (20.75%)	26 (21.49%)	61 (22.02%)			
Unknown	8 (14.55%)	7 (13.21%)	26 (21.49%)	52 (18.77%)			
**New Neoplasm Event Post Initial Therapy Indicator**					0.517	0.869	0.906
Yes	16 (29.09%)	17 (32.08%)	43 (35.54%)	95 (34.30%)	
No	31 (56.36%)	29 (54.72%)	64 (52.89%)	146 (52.71%)	
Unknown	8 (14.55%)	7 (13.21%)	14 (11.57%)	36 (13.00%)	

Data are n (%) or median (range). Bold values represent statistical differences. ^†^Determined by Mann-Whitney test. Other statistical comparisons between groups were made by Fisher exact test.

### The Subgroup of Lung Adenocarcinoma With Co-Mutation of FAT3 and LRP1B Exhibited Favorable Immunotherapy Efficacy

**Figure 4:**
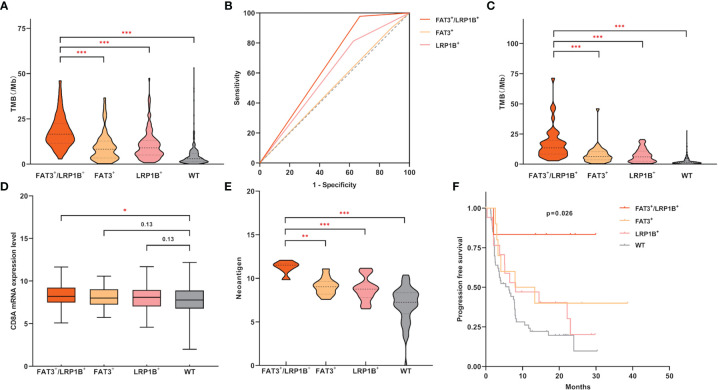
LUAD samples with FAT3 and LRP1B co-mutation had significantly higher TMB levels and exhibited unique immune characteristics. **(A)** Compared with LUADs with only FAT3 or LRP1B mutations alone, the LUAD subgroup with co-mutation of FAT3 and LRP1B had significantly higher TMB level. **(B)** High specificity of FAT3 and LRP1B co-mutation status for predicting TMB-H (>10 mutations/Mb). The prediction performance of the FAT3^+^/LRP1B^+^ group for TMB-H exceeded that of the FAT3^+^ and LRP1B^+^ groups, with an AUC of 0.655, a 95% confidence interval of 0.596-0.713, and p<0.001. **(C)** The validation cohort of 485 LUAD samples confirmed the FAT3^+^/LRP1B^+^ group had the highest TMB level. **(D)** Compared with wild-type samples, LUADs in the FAT3^+^/LRP1B^+^ group had significantly higher CD8A mRNA expression level (adding one pseudo-count and log_2_ transformation). **(E)** The level of neoantigen (adding one pseudo-count and log2 transformation) in the FAT3^+^/LRP1B^+^ group was higher than that in the FAT3^+^ and LRP1B^+^ groups. **(F)** The immunotherapy PFS of the FAT3^+^/LRP1B^+^ group was significantly prolonged. Statistical comparisons between different groups were made by Mann-Whitney test **(A, C–E)**, and survival curves were plotted using the Kaplan-Meier method and compared by the log-rank test **(F)**. *p < 0.05, **p < 0.01, ***p < 0.001.

Considering the co-occurrence of FAT3 and LRP1B mutations, as well as the unique clinical characteristics of the co-mutation group, we attempted to check whether the molecular features of the FAT3^+^/LRP1B^+^ group were also worthy of attention. We found that LUADs in the FAT3^+^/LRP1B^+^ group had the highest level of TMB, even significantly higher than that of the FAT3^+^ (**Figure 4A**, 16.5 vs 8.3, p<0.001) and LRP1B^+^ (**Figure 4A**, 16.5 vs 9.0, p<0.001) groups. To evaluate the accuracy of using FAT3 and LRP1B co-mutation to predict TMB level in LUAD, 10 mutations/Mb was regarded as the cut-off value of TMB like most studies (5, 8). The positive rate of high TMB (>10 mutations/Mb) among the FAT3^+^/LRP1B^+^ group was 85.45% (47 of 55), while that of the remaining samples of LUAD cohort was 21.06% (95 of 451), p<0.001 (Fisher exact test). Besides, to determine the sensitivity and specificity of FAT3 and LRP1B co-mutation for predicting the level of TMB, receiver operating characteristic (ROC) curve was performed. The area under the ROC curve (AUC) of co-mutation group was 0.655, with a sensitivity of 33.1% and a specificity of 97.8% (**Figure 4B**, p<0.001). The AUC of the FAT3^+^/LRP1B^+^ group was higher than that of the FAT3^+^, LRP1B^+^ and WT groups, illustrating that the FAT3 and LRP1B co-mutation had superior predictive performance for TMB level. What’s more, another dataset composed of 485 LUAD samples was used as the validation group, further supporting the view that the TMB level of the co-mutation group was higher than that of the FAT3 and LRP1B groups (**Figure 4C**). At the same time, although the previous result indicated that the population with FAT3 and LRP1B mutations had higher CD8A expression level than wild-type LUADs, a more detailed group comparison result proved that compared with the WT group, the CD8A expression level of the FAT3^+^/LRP1B^+^ group was significantly up-regulated, while not the same case in the FAT3^+^ and LRP1B^+^ groups (**Figure 4D**).

Similarly, through a population of 90 LUAD patients that received ICIs treatment, we discovered that the FAT3^+^/LRP1B^+^ group had strikingly higher neoantigen level than the FAT3^+^ (**Figure 4E**, p<0.01) and LRP1B^+^ (**Figure 4E**, p<0.001) groups. Even more importantly, LUAD patients with co-mutation of FAT3 and LRP1B had significantly better immunotherapy outcomes (**Figure 4F**, p=0.026). Moreover, when the entire cohort was divided into two groups, only the co-mutation group exhibited significantly prolonged PFS, while the immunotherapy response of the FAT3 and LRP1B single-mutation groups showed no advantage (**Supplementary Figure 2**). Taken together, these data suggest that the co-mutation of FAT3 and LRP1B defines a unique subset and may be a promising novel biomarker for screening candidates for ICIs therapy of lung adenocarcinoma.

### Pathway Enrichment Analysis of FAT3 and LRP1B Co-Mutation

**Figure 5:**
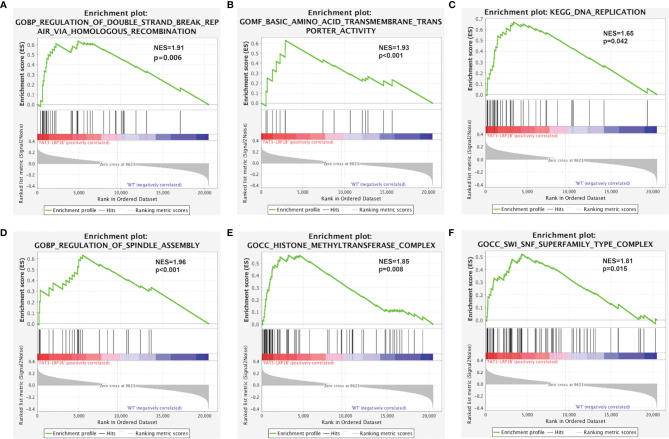
Significantly enriched pathways associated with LUAD samples with co-mutations of FAT3 and LRP1B. GSEA analysis revealed that the FAT3^+^/LRP1B^+^ subgroup significantly enriched in **(A)** Regulation of double strand break repair *via* homologous recombination, **(B)** Basic amino acid transmembrane transporter activity, **(C)** DNA replication, **(D)** Regulation of spindle assembly, **(E)** Histone methyltransferase complex, **(F)** SWI/SNF superfamily type complex. NES, normalized enrichment score.

Gene set enrichment analysis was performed with the TCGA LUAD dataset to explore the effects of FAT3 and LRP1B co-mutation on the physiological processes and functions of the body. The analysis results of GSEA indicated that LUADs with FAT3 and LRP1B co-mutation were significantly enriched in Regulation of double strand break repair *via* homologous recombination, Basic amino acid transmembrane transporter activity, DNA replication, Regulation of spindle assembly, Histone methyltransferase complex and SWI/SNF superfamily type complex pathways (**Figure 5**), which revealed the potential mechanism of co-mutation in the occurrence and development of LUAD tumors, and also provided a direction for subsequent further research.

### Co-Mutation of FAT3 and LRP1B Affects Lymphocyte Infiltration

**Figure 6:**
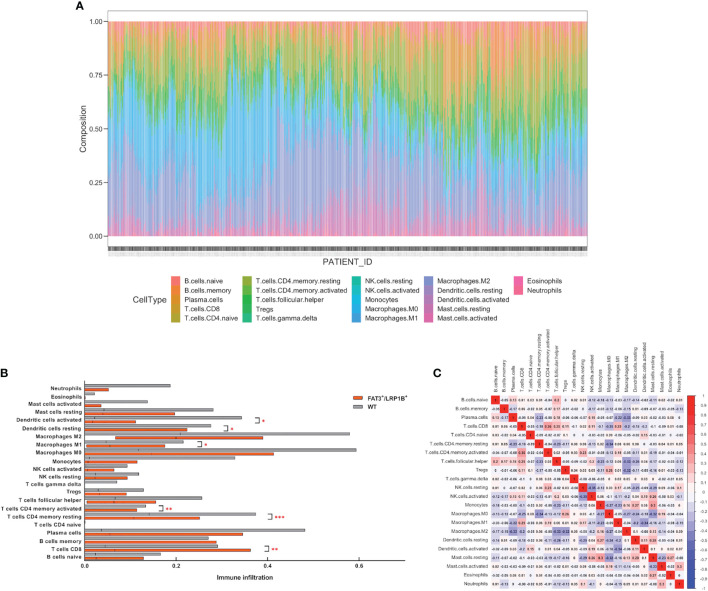
Co-mutation of FAT3 and LRP1B can affect the degree of lymphocyte infiltration in LUAD. **(A)** The proportion of 22 immune cells in each sample in the LUAD dataset. **(B)** Differential tumor-infiltrating immune cells between FAT3^+^/LRP1B^+^ group and wild-type group. **(C)** Correlation analysis of immune cell abundance in tumor microenvironment. Red represents positive correlation, blue represents negative correlation. p values indicate comparisons between FAT3^+^/LRP1B^+^ and wild-type LUADs by Mann-Whitney test. *p < 0.05, **p < 0.01, ***p < 0.001.

The efficacy of ICIs treatment in cancer patients has been linked to the quality and magnitude of tumor-infiltrating lymphocytes (TILs) within the microenvironment (39). The infiltration degree of 22 immune cells in the LUAD was calculated using the CIBERSORT algorithm, and the proportion of immune cells in each sample in the cohort was displayed through stacked bars (**Figure 6A**). The results showed that compared with the wild-type samples, the TME of the LUADs with co-mutation of FAT3 and LRP1B contained more CD8^+^ T cells, activated CD4^+^ memory T cells and M1 macrophages, but fewer resting CD4^+^ memory T cells, resting dendritic cells and activated dendritic cells (**Figure 6B**). Besides, Pearson correlation analysis of immune cell abundance revealed the feedback relationship between immune cells (**Figure 6C**). From the correlation matrix, the strongest positive correlation existed between CD8^+^ T cells and activated CD4^+^ memory T cells, while the negative correlation between activated NK cells and resting NK cells was the strongest.

## Discussion

Based on a TCGA dataset consisting of 506 samples, we conducted an integrated analysis of the somatic mutation landscape of lung adenocarcinoma. After stepwise screening, we obtained FAT3 and LRP1B, two key genes closely related to the immunogenicity, cytotoxicity and immunotherapy response of lung adenocarcinoma patients. Further analysis showed that there was a co-occurrence relationship between FAT3 and LRP1B mutations, so we took the LUAD subgroup with FAT3 and LRP1B co-mutation as a whole to conduct a more in-depth exploration. The results demonstrated that the frequency of co-mutation of FAT3 and LRP1B in LUAD patients was 10.87% (55 out of 506), which was associated with an earlier age of onset and occurred more frequently in Black/African American. Besides, LUAD patients with FAT3 and LRP1B co-mutation displayed substantially higher TMB, CD8A expression level and neoantigen. More importantly, the subset of LUAD with co-mutation of FAT3 and LRP1B exhibited significantly prolonged immunotherapy PFS, so we performed GSEA and immune infiltration analysis on this subset to explore the effect of co-mutation on the degree of lymphocyte infiltration and tumorigenesis in lung adenocarcinoma.

FAT3 mutations occurred in 21.34% (108 out of 506) of LUAD samples in the TCGA cohort, which is consistent with previous conclusions in TNBC and ESCA that FAT3 gene has higher mutation rate (32, 33). Similarly, LRP1B gene not only has a relatively high mutation frequency (34.78%, 176/506) in this study, but also frequently mutates in multiple tumors, such as melanoma, ESCA and colorectal cancer (CRC) (28). Previous studies have demonstrated that in a variety of solid tumors, samples with FAT3 (31–33) or LRP1B (28, 29) mutations may have elevated TMB. However, according to our data, we found that although the LUAD samples with a single mutation of FAT3 or LRP1B had higher levels of TMB than the wild-type group, the TMB level of LUADs with co-mutation of FAT3 and LRP1B was even significantly higher than that of the FAT3 and LRP1B single-mutation groups. The increased TMB level of the co-mutation group was also reflected in its elevated neoantigen level, which was higher than that in the FAT3^+^ and LRP1B^+^ groups. Higher neoantigen load represents enhanced immunogenicity, which can stimulate more potent cytotoxicity, and our research suggests that only the FAT3^+^/LRP1B^+^ group has significantly increased CD8A expression level, while the FAT3 or LRP1B single-mutation groups have no significant difference from the wild-type group. It is worth noting that although the subset with FAT3 or LRP1B mutations displayed superior immunotherapy PFS than the wild-type, the results of a more detailed study of the cohort based on the co-mutation status indicated that only LUAD patients with co-mutation of FAT3 and LRP1B receive ICIs treatment can achieve better efficacy. Taken together, our study proves that while a series of studies have confirmed the predictive role of FAT3 and LRP1B mutations in tumor immunotherapy, the results of genetic testing found that LUAD patients with FAT3 or LRP1B mutations may not necessarily benefit from immunotherapy, only the LUAD population with both FAT3 and LRP1B mutations can benefit from ICIs therapy.

GSEA results showed that multiple signaling pathways were enriched in the LUAD subset with co-mutation of FAT3 and LRP1B. Basic amino acid transmembrane transporter activity enables the transfer of basic amino acids from one side of a membrane to the other, thereby providing nutrients for human metabolism. Most of the remaining enriched pathways are related to cell cycle and transcriptional regulation, illustrating that co-mutation may affect the occurrence and development of tumors through these mechanisms. Strikingly, the DNA homologous recombination repair (HRR) pathway is also enriched in the FAT3^+^/LRP1B^+^ group. Homologous recombination deficiency (HRD) leads to the accumulation of a large amount of DNA with damage that cannot be repaired, thus increasing genomic instability, which may be one of the reasons for the higher TMB level of LUADs with co-mutation of FAT3 and LRP1B. Studies have shown that NSCLC patients with HRD have better response to ICIs treatment (40, 41), which further validates the viewpoints put forward in this study.

The immune system and tumor microenvironment play an important role in tumor growth and progression, and can contribute to the efficacy of immunotherapy (42). Immune cells are an important component of the tumor microenvironment. We found that compared with wild-type samples, LUADs with co-mutation of FAT3 and LRP1B displayed a higher degree of infiltration of CD8^+^ T cells, activated CD4^+^ memory T cells and M1 macrophages. Cytotoxic CD8^+^ T cells (CTLs) are the most powerful effectors in the anti-cancer immune response, because they can detect intracellular antigens presented by major histocompatibility complex (MHC), which constitute the backbone of cancer immunotherapy (43). CD4^+^ T cells can help enhance the function of CTLs, enabling CTLs to overcome the barriers that typically hinder anti-cancer immunity (44). And M1 macrophages promote inflammatory response and play an active role in the elimination of pathogens and tumor cells (45). ICIs therapy can induce polarization of M1 macrophages, thus enhancing the antineoplastic effect (46, 47). The simultaneous prominence of the three types cells, CD8^+^ T cells, activated CD4^+^ memory T cells and M1 macrophages, implying that co-mutation of FAT3 and LRP1B can be used to predict the cytotoxic effect of lymphocytes against tumor cells and the beneficial response of immunotherapy in LUAD.

Our study also has some limitations. In view of the retrospective nature of this study that did not collect real-world samples, the conclusions obtained need to be verified by a large-sample prospective analysis. In addition, the cohort used for mutation and expression analysis is completely different from the population that has received immunotherapy, making our findings may need to be interpreted with caution. Nevertheless, we believe that the data of this paper has proved that in patients with lung adenocarcinoma, the co-mutation status of FAT3 and LRP1B deserves further attention and research. Considering that FAT3 and LRP1B genes lack hotspot mutations in lung adenocarcinoma, and these two genes may not even be included in the list of clinical routine gene testing (especially FAT3), we recommend that whole exon or even the whole gene level mutations of FAT3 and LRP1B should be comprehensively detected in clinical practice, which will provide more useful information for treatment options of lung adenocarcinoma.

In conclusion, through a comprehensive analysis of lung adenocarcinoma samples, the results of this study provide insights into the immunotherapeutic implications of co-occurrence of some common mutations in lung adenocarcinoma. We found that co-mutation of FAT3 and LRP1B has prominent significance, which can increase somatic mutational load, boost cytotoxicity and tumor immunogenicity, facilitate lymphocyte infiltration in the microenvironment, and significantly influence the outcome of immunotherapy. This research provides evidence that co-mutation of FAT3 and LRP1B may be a very promising novel biomarker for screening candidates of ICIs therapy in lung adenocarcinoma and lays a preliminary foundation for subsequent further exploration.

## Data Availability Statement

Publicly available datasets were analyzed in this study. This data can be found here: The Cancer Genome Atlas (TCGA, http://cancergenome.nih.gov).

## Author Contributions

MZ, RJ, and ZJ conceived and designed the study. MZ and LZ performed the study. MZ, LZ, HC, QZ, HW, and BZ analyzed the result. MZ, LZ, RJ, and ZJ wrote the paper. All authors reviewed the manuscript and approved the submitted version.

## Funding

This work was supported by grants from the National Natural Science Foundation of China (No. 81803914), Tianjin Cancer Hospital College-level Research Seed Fund (No. 1810) and Health Science and Technology Project of Tianjin City of China (No. RC20189).

## Conflict of Interest

The authors declare that the research was conducted in the absence of any commercial or financial relationships that could be construed as a potential conflict of interest.

The reviewer SX declared a shared affiliation with the authors to the handling editor at the time of review.

## Publisher’s Note

All claims expressed in this article are solely those of the authors and do not necessarily represent those of their affiliated organizations, or those of the publisher, the editors and the reviewers. Any product that may be evaluated in this article, or claim that may be made by its manufacturer, is not guaranteed or endorsed by the publisher.
