# Supplementary material for: Co-Mutation of FAT3 and LRP1B in Lung Adenocarcinoma Defines a Unique Subset Correlated With the Efficacy of Immunotherapy
Source: Front Immunol. 2022 Jan 6;12:800951. doi: 10.3389/fimmu.2021.800951 (PMC8770854; doi:10.3389/fimmu.2021.800951)
Supplement: Supplementary file 1 [file DataSheet_1.docx]

Supplementary Material

**Supplementary Figures**

**
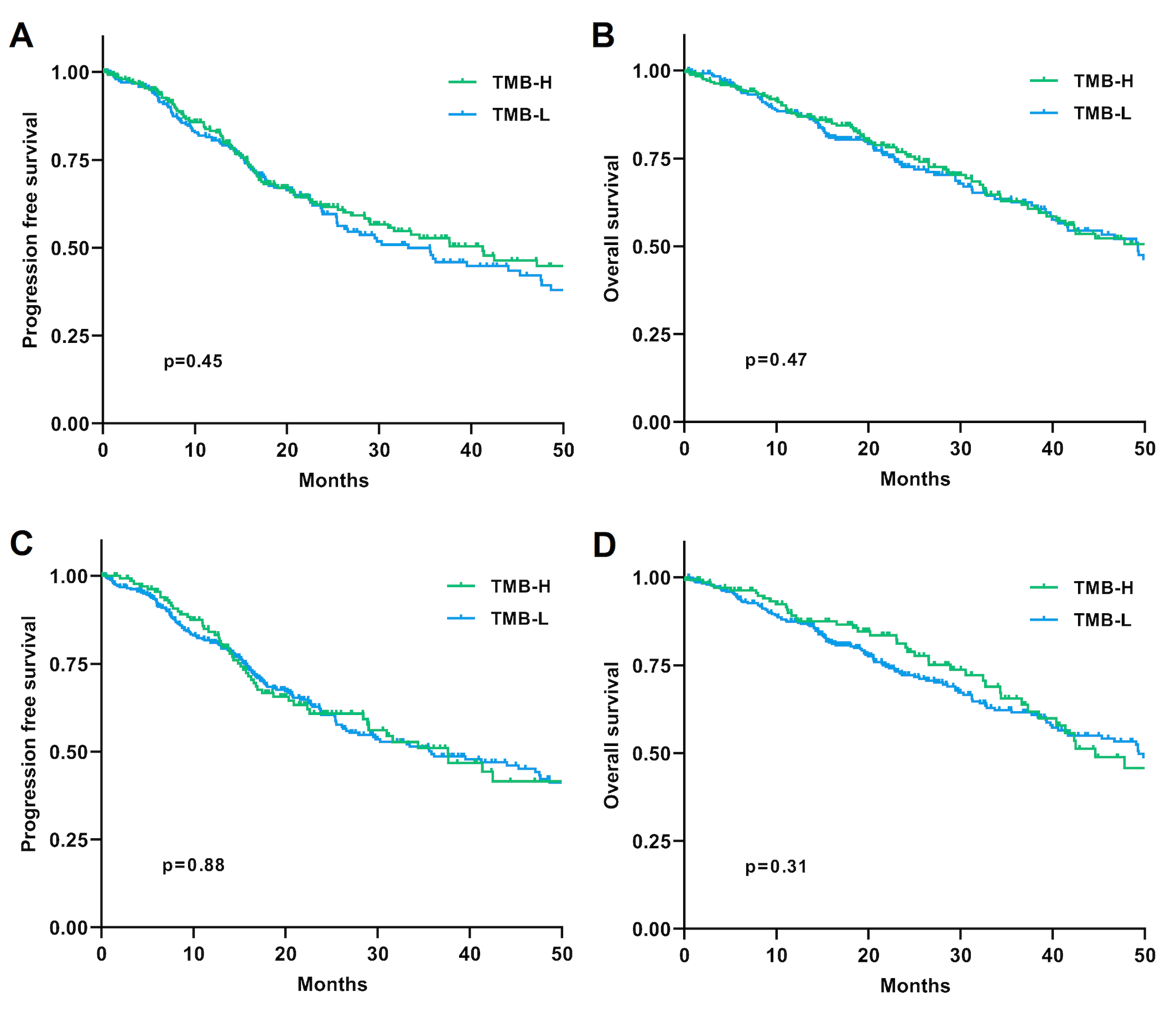
Supplementary Figure 1.** There was no significant correlation between TMB level and prognosis in LUAD. **(A)**-**(B)** Using median TMB as the cut-off value, the progression free survival and overall survival of TMB-H and TMB-L in the LUAD cohort. **(C)**-**(D)** Progression free survival and overall survival of TMB-H and TMB-L of the LUAD dataset when TMB levels were divided by 10 mutations/Mb. Survival curves were plotted using the Kaplan-Meier method and compared by the log-rank test.


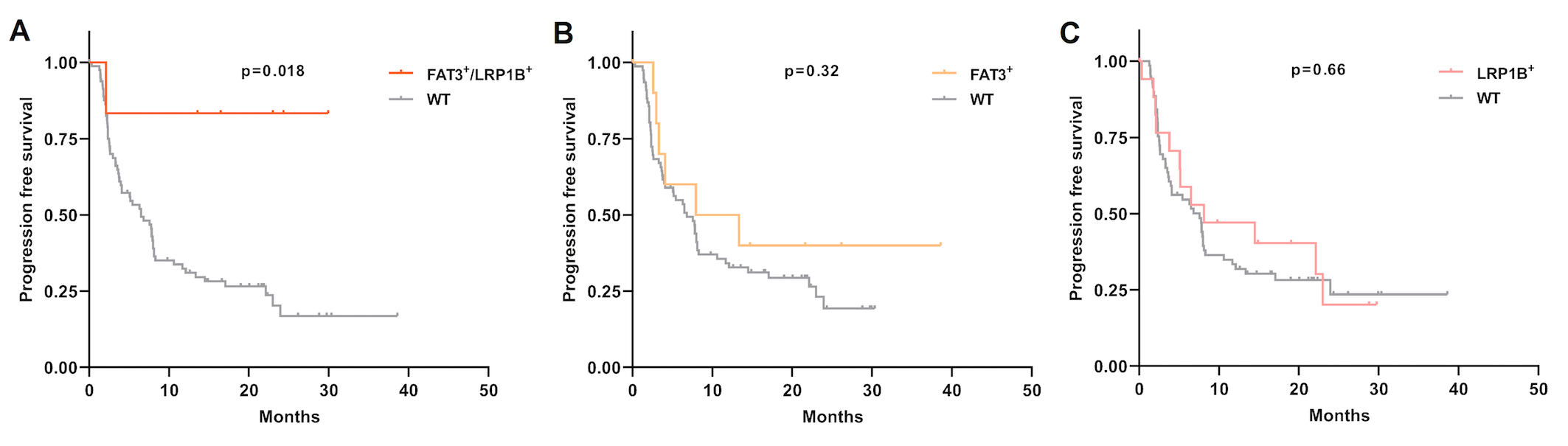
**Supplementary Figure 2.** Clinical outcomes of LUADs according to FAT3 and LRP1B mutation status. **(A)** Compared with wild-type, LUADs with FAT3 and LRP1B co-mutations had significantly prolonged immunotherapy PFS. **(B)**-**(C)** LUADs with only FAT3 or LRP1B mutations showed no significant differences in immunotherapy PFS compared to wild-type samples. p values indicated comparisons between mutant and wild-type LUADs by unadjusted log-rank test.
